# Normal values for pancreatic stone protein in different age groups

**DOI:** 10.1186/s12871-015-0149-y

**Published:** 2015-11-20

**Authors:** Luregn J Schlapbach, Eric Giannoni, Sven Wellmann, Martin Stocker, Roland A Ammann, Rolf Graf

**Affiliations:** 1 Mater Research Institute, Paediatric Critical Care Research Group, University of Queensland, Brisbane, Australia; 2 Paediatric Intensive Care Unit, Lady Cilento Children’s Hospital, Children’s Health Queensland, South Brisbane, QLD 4101 Australia; 3 Department of Pediatrics, University of Bern, Bern, Switzerland; 4 Service of Neonatology, Centre Hospitalier Universitaire Vaudois and University of Lausanne, Lausanne, Switzerland; 5 Infectious Diseases Service, Centre Hospitalier Universitaire Vaudois and University of Lausanne, Lausanne, Switzerland; 6 Department of Neonatology, University Children’s Hospital Basel, Basel, Switzerland; 7 Neonatal and Pediatric Intensive Care Unit, Children’s Hospital Lucerne, Lucerne, Switzerland; 8 Department of Surgery, Swiss HPB Center, University Hospital Zurich, Zurich, Switzerland

**Keywords:** Intensive care unit, Pancreatic stone protein, Sepsis

## Abstract

**Background:**

Pancreatic stone protein (PSP) has been identified as a promising sepsis marker in adults, children and neonates. However, data on population-based reference values are lacking. This study aimed to establish age-specific reference values for PSP.

**Methods:**

PSP was determined using a specific ELISA. PSP serum concentrations were determined in 372 healthy subjects including 217 neonates, 94 infants and children up to 16 years, and 61 adults. The adjacent categories method was used to determine which age categories had significantly different PSP concentrations.

**Results:**

PSP circulating levels were not gender-dependent and ranged from 1.0 to 99.4 ng/ml with a median of 9.2 ng/ml. PSP increased significantly between the age categories, from a median of 2.6 ng/ml in very preterm newborns, to 6.3 ng/ml in term newborns, to 16.1 ng/ml in older children (*p* < 0.001). PSP levels were higher on postnatal day three compared to levels measured immediately post delivery (*p* < 0.001). Paired umbilical artery and umbilical vein samples were strongly correlated (*p* < 0.001). Simultaneously obtained capillary heel-prick versus venous samples showed a good level of agreement for PSP (Rho 0.89, bias 19 %).

**Conclusions:**

This study provides age-specific normal values that may be used to define cut-offs for future trials on PSP. We demonstrate an age-dependent increase of PSP from birth to childhood.

## Background

In recent years, a number of biomarkers have been investigated as candidates to improve the ability to diagnose infection and sepsis in newborns, children and adults [1–6]. A limitation of several recently proposed infection markers is the lack of larger cohorts providing populational serum concentration measurements in order to establish normal reference values. This is important because the performance of most biomarkers is context-sensitive and in some cases age-specific. For example, C-reactive protein is increased after surgical interventions [7], which reduces its specificity to diagnose infections in postoperative patients. Procalcitonin (PCT) is physiologically elevated during the first 48 h of life, which has to be taken into account when defining optimal cutoffs for the diagnosis of neonatal early-onset sepsis [6].

Pancreatic stone protein (PSP) has recently emerged as a promising marker of sepsis [8, 9]. PSP has so far been studied predominantly in adults, and was shown to predict multi-organ failure and mortality in patients with ventilator-associated pneumonia and post-traumatic sepsis [8, 9]. A study using a bioscore combinding PSP with procalcitonin (PCT) significantly improved the ability to diagnose neonatal early-onset sepsis, with a negative predictive value of 100 % if both PSP and PCT were negative [10]. These data suggest that PSP is a promising candidate for further studies assessing performance and use in clinical practice. To date, however, no reference values are available for PSP. While its main physiological role is poorly understood, PSP may act by promoting cellular proliferative responses in the pancreas and was shown to activate polymorphonuclear cells [9]. Animal studies have shown induction of PSP expression in subsets of intestinal and gastric cells by stress conditions in the absence of direct pancreatic inflammation [11].

The present study aimed to determine circulating PSP serum levels in healthy individuals from birth to adulthood in order to provide reference values for clinical use and future studies.

## Methods

### Study subjects

Subjects for this study were recruited through three different studies conducted between July 1^st^ 2009 and December 31^st^ 2012. Study subjects were eligible if they did not present with any infection or major disease at time of sampling, and if subsequent investigation for infection resulted negative. The study was submitted to and approved by the institutional ethics committees at each of the involved hospitals (Cantonal Ethics Committees of the Canton of Berne, Canton of Zurich and Canton of Lucerne, and Bicantonal Ethics Committee of Basel, Switzerland). Written consent from the parents of pediatric participants, and from adult study participants was obtained.

104 patients included in this study were enrolled through the prospective, randomized-controlled multicentre Neonatal Procalcitonin Intervention Study (NeoPInS, see http://www.clinicaltrials.gov, trial number NCT00854932) [12], in three Swiss neonatal intensive care units (Department of Neonatology, University Children’s Hospital Bern and Children’s Hospital Lucerne; Department of Pediatrics, Cantonal Hospital Winterthur, Switzerland). Children were included in the present study only if they were admitted within the first 72 h of life to the Neonatal Intensive Care Unit for suspicion of early-onset sepsis that was subsequently ruled out. Infection was ruled out using all of the following criteria: 1. Negative cultures, 2. Normal infection markers, 3. No signs of systemic inflammatory response syndrome (SIRS). Newborns with complex congenital malformations, chromosomal aberrations, and congenital heart disease and those who died within the first week after admission were excluded.

Umbilical cord blood samples were obtained in 113 patients. Umbilical cord samples paired with postnatal samples were obtained prospectively at the University Hospital of Zurich, Switzerland, between August 2011 and April 2012 in preterm and term newborns in the absence of major neonatal disease, infections or comorbidities [13]. Umbilical cord blood was collected from the umbilical artery (UA) and the umbilical vein (UV) after delivery of the placenta. Peripheral blood was then collected on day of life three in 22 infants.

Venous samples in the pediatric age groups were recruited through a prospective study in 94 children (aged between >1 month and 16 years) admitted for elective surgery at the Department of Pediatrics, University of Lausanne, Switzerland, between April 26, 2012, and December 20, 2012. Infants and children with acute or chronic infection, severe underlying disease or chromosomal abnormality were excluded from the study.

Venous samples from adults were recruited through healthy adult volunteers (*N* = 61).

### Blood sampling

In each patient, 100 μl of blood was sampled. Samples were centrifuged directly after sampling for 6 min at 3000 G, the serum was then frozen within less than 4 h in sterile tubes at −80 °C until measurement. Venous blood was taken by venipuncture from a peripheral vein.

PSP was measured in simultaneous samples obtained in the umbilical artery and the umbilical vein in 21 infants. Cord blood was collected immediately after delivery of the child from the umbilical vein at the placental side of the cord.

In 20 newborns, 100 μl of capillary and 100 μl of venous blood were sampled simultaneously [14]. Venous blood was taken by venipuncture from a peripheral vein using IV catheters (Insyte-N™ 24G, Becton Dickinson AB, Helsingborg, Sweden). Capillary blood was taken by heel prick with an automated lancet (Tenderfoot preemie®, ITC, Edison, New Jersey, USA). The thereby collected tubes (S-Monovette®, and Microvette 100®, Sarstedt AG & Co., Nümbrecht, Germany) were centrifuged directly after sampling for 6 min at 3000 G, the serum was then immediately frozen in sterile tubes at −80 °C.

### Pancreatic stone protein (PSP) measurements

PSP was analyzed using a specific ELISA as previously described [9]. In brief, patient serum was incubated with guinea pig anti-human PSP/reg antibody precoated plates. Rabbit anti-human PSP/reg was added and subsequently detected by phosphatase-conjugated anti-rabbit IgG. The detection limit was 0.1 ng/mL and the interplate variance was <10 %.

### Statistical methods

In the figures displaying PSP serum concentrations vs. age, logarithmized scales were used. To cover the wide age range from preterm and term neonates over children to adults, Age_Graphics_ calculated in years from week 0 gestational age (wGA) was used here. The specific calculations were Age_Graphics_ = wGA/52 for neonates, and Age_Graphics_ = age in years + 40/52 for children and adults. The age-dependent estimate of the mean and its 95 % confidence interval were calculated by fourth degree linear regression. The adjacent categories method, applying the Wilcoxon– Mann–Whitney test, was used to determine which age categories had significantly different PSP concentrations as described [15]. It started with 12 categories defined by 11 age limits set at <32 and <37 (preterm neonates), <40, and <42 wGA (term neonates), <1 year (infants), <4, <8, <12, and <16 years (children and adolescents), and <30 and <50 years (adults). Then, adjacent age categories with the least significant differences were combined step by step until concentrations were significantly different between the resulting combined age categories. For these categories, the minimum, the 10th percentile (P10), P25, the median (P50), P75, P90, the maximum, and the number and proportion of results ≤ P10 in adults and ≥ P90 in adults were calculated.

Bland-Altman plots were constructed, and the mean bias and the 95 % limits of agreement were calculated based on logarithmized concentrations.

Two-sided tests were used throughout, and *p*-values <0.05 were considered statistically significant. The software used was R 3.0.2 (R Foundation for Statistical Computing, Vienna, Austria), SPSS 18.0 (SPSS Inc., Chicago, Illinois, USA), and Prism 5.0c (GraphPad Software, La Jolla, California, USA).

## Results

PSP serum concentrations were determined in 372 healthy subjects including 217 neonates, 94 infants and children up to 16 years; and 61 adults. Neonatal samples included cord blood samples from 113 infants born between 27.6 and 41.9 weeks gestational age (median gestational age 39 weeks, IQR 35.1 to 40.3 weeks), and postnatal samples in 104 newborns born between 34.0 and 42.0 weeks (median gestational age 39 weeks, IQR 35.5 to 40.2 weeks). Control samples in adults were received from volunteers of a Caucasian population with a median age of 45 years (19–82). There was no gender difference in the PSP values in adults.

**Fig. 1:**
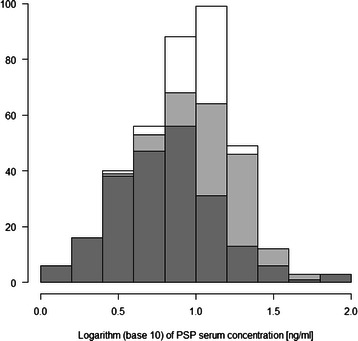
Histogram of logarithmically transformed Pancreatic Stone Protein (PSP) serum concentrations in 372 healthy subjects (dark grey, 217 neonates; light grey, 94 infants and children up to 16 years; white, 61 adults above 16 years)

**Fig. 2:**
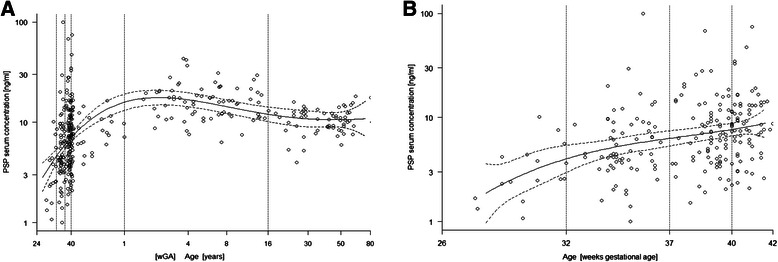
Serum concentration of Pancreatic Stone Protein (PSP) vs. age in **a**) 372 healthy subjects, and **b**) 217 healthy neonates. The solid line indicates the mean predicted by linear regression with its 95 % confidence interval. The dotted lines indicate limits between age categories with significantly different serum concentration

PSP ranged from 1.0 to 99.4 ng/ml with a median of 9.2 ng/ml. PSP was non-normally distributed (Shapiro-Wilks test statistic, W = 0.6793; *p* < 0.001), whereas logarithmically transformed PSP concentrations were approximately normally distributed (W = 0.989; *p* = 0.007; Fig. 1). Figure 2 shows the bell-shaped distribution of PSP from birth to adulthood. PSP increased significantly between the age categories, from a median of 2.6 ng/ml in very preterm newborn, to 4.5 ng/ml in preterm newborn, to 6.3 ng/ml in term newborn before 40 weeks gestational age, to 8.4 ng/ml in term newborn after 40 weeks gestational age and infants, and to 16.1 ng/ml in older children. Values in adults were significantly lower than in children with a median of 10.8 ng/ml (*p* < 0.001), but still higher than in newborns (Table 1). PSP serum levels in cord blood were higher in infants born with perinatal acidosis (Spearman’s Rho −0.25, *p* < 0.001). PSP levels were not significantly different between male and female study subjects (Mann–Whitney Z −1.68, *p* = 0.094). Delivery mode (cesarean versus vaginal) was not associated with PSP levels (Z −0.059, *p* = 0.95).

**Table 1:** Serum concentration of pancreatic stone protein in ng/mL

Age category	*N*	Minimum	P10	P25	Median	P75	P90	Maximum	*N* (%) ≤ P10_adult_	*N* (%) ≥ P90_adult_
<32 wGA	15	1.1	1.4	2.0	2.6	4.3	6.8	10.1	14 (93 %)	0 (0 %)
32 wGA to 36 wGA	62	1.0	2.2	3.5	4.5	8.4	12.1	99.4	45 (73 %)	4 (6 %)
37 wGA to 39 wGA	71	1.5	3.0	4.0	6.3	10.2	16.5	68.0	37 (52 %)	11 (15 %)
40 wGA to <1 year	89	2.1	3.7	5.9	8.4	12.5	16.4	74.4	37 (42 %)	11 (12 %)
1 year to <16 years	74	6.3	10.3	12.0	16.1	20.2	25.0	43.0	4 (5 %)	45 (61 %)
Adults	61	4.0	7.6	9.0	10.8	12.5	14.5	18.3	7 (11 %)	7 (11 %)

*P* Percentile, *wGA* Weeks gestational age, *yr* year

**Fig. 3:**
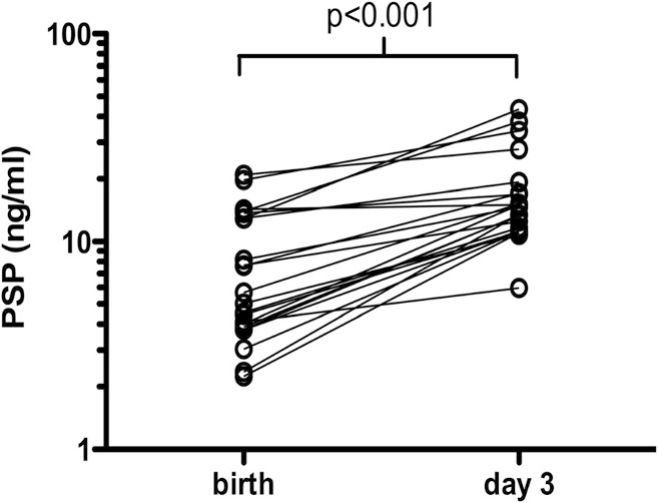
Postnatal increase of Pancreatic Stone Protein (PSP). Paired PSP determinations in cord blood (birth) and on the third day of life (day 3) are shown (*n* = 22). The *p*-value of the Wilcoxon signed rank test is given

In order to assess changes of PSP during the first days of life, PSP was measured sequentially in cord blood and on day 3 of life in 22 patients. Cord blood and postnatal samples were positively correlated (Rho 0.73, *p* < 0.001). PSP showed a significant postnatal increase (median 14.1 versus 5.4 ng/ml, *p* < 0.001), see Fig. 3.

We tested whether the site of blood collection had an influence by comparing the values from umbilical vein versus umbilical artery, and capillary heel prick versus venous blood. Paired umbilical artery and umbilical vein samples were strongly correlated (*N* = 21, Spearman’s Rho 0.96, *p* < 0.001). Correspondingly, there was not a relevant difference between umbilical vein versus umbilical arterial PSP concentrations (*p* = 0.50).

**Fig. 4:**
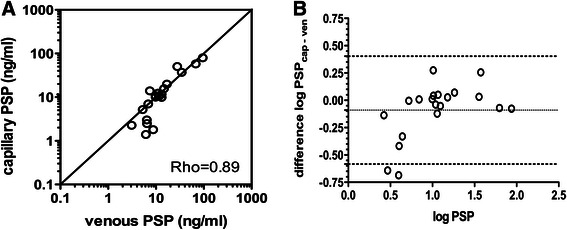
Comparison of capillary and venous Pancreatic Stone Protein (PSP) concentrations. **a** Scatter plot comparing capillary and venous PSP concentrations. The bisecting line is shown (straight line, indicating 100 % agreement). The correlation coefficient of the Spearman’s rank test (Rho) is given. **b** Bland-Altman plot showing the difference between logarithmically transformed capillary to venous Pancreatic Stone Protein (PSP) concentration plotted against the average of capillary and venous concentration. Bias (mean difference, dotted line) and 95 %-limits of agreement (dashed lines) are shown. The units shown indicate logarithms (base 10) of PSP concentrations (ng/ml)

PSP concentrations were then compared between simultaneously obtained capillary heel-prick versus venous samples with a good level of agreement (*n* = 20, Rho 0.89, bias 19 %, Fig. 4a and b). The limits of agreement between capillary and venous samples for PSP were comparable to the calculated limits of agreement based on the coefficients of variation of the assay.

## Discussion

We report on age-specific serum concentrations in PSP in healthy individuals. The results indicate that PSP shows a relevant age-dependent variation, with lowest values observed in premature newborns, followed by a rise until childhood and adolescence. We observed a slight decrease of PSP serum concentrations in adults in comparison to children and adolescents. We observed a significant increase of PSP with increasing gestational age, and a more than twofold postnatal rise.

PSP has predominantly been studied in critically ill adults, with highest levels found in patients with sepsis and multi-organ failure [1, 8, 9]. In recent ICU based studies, high PSP levels predicted mortality in septic adults, whereas established infection markers such as CRP or PCT were increased in presence of infections, but did not allow to stratify for sepsis mortality risk [16, 17]. In a study on critically ill children, PSP levels were significantly higher in patients with multiorgan dysfunction syndrome, and patients who died tended to have higher PSP levels accordingly [18]. Similarly, increased PSP levels were observed in newborns with early-onset sepsis [10]. The performance of PSP was comparable or superior to other markers such as CRP, PCT or soluble human triggering receptor expressed on myeloid cells-1 (sTREM-1), suggesting PSP is independently increased in presence of infections. In addition, studies performed in ICUs indicate that significant PSP increase occurs in presence of significant stress and multi-organ failure [1, 8, 9].

Previous publications investigating PSP in sepsis and shock defined optimal cutoff thresholds using receiver-operating characteristics curve analyses based on the respective study cohorts. Notably, these resulted in different cutoffs for PSP, which were mostly higher than the 90^th^ centile for PSP in the populational reference patients in our cohort. For example, in newborns with early-onset sepsis, a PSP cutoff of 9 ng/ml discriminated best from non-infected infants [10]. In adult patients with chronic obstructive pulmonary disease, PSP cutoff values of 33.9 ng/mL combined with the presence of discolored sputum had a specificity of 97 % to identify patients with pathogenic bacteria on sputum culture [19], and predicted exacerbations. PSP >130 ng/ml predicted mortality in adults with peritonitis [17]. Of note, adult studies in patients with sepsis reported serum concentrations as high as >1000 ng/ml [17]. In view of the considerable variability in cutoffs used by previous studies, the present study fills an important gap by providing reference ranges for future studies [20]. In practice, different thresholds for PSP may need to be applied in newborns compared to older age groups.

Of note, the inter-individual variability of PSP was highest in the umbilical cord values, with a range from 1 to 99 ng/ml, and PSP was increased in infants with perinatal acidosis. These findings may suggest that the stress response associated with birth can increase PSP.

Interestingly, a large number of serum proteins show a physiologic increase with gestational age followed by rapid postnatal increase [21]. The precise mechanisms are unclear, and likely relate to a transient physiological systemic inflammatory response due to the microbiological colonization of skin and mucosal surfaces, including gut exposure to microbiota [22].

Strengths of the present study include the large sample size capturing a wide range of age span from extremely preterm infants to older adults, and the restriction of samples to individuals in the absence of with disease or infection. The good agreement between capillary and venous PSP samples observed suggests that capillary sampling for PSP is accurate, which may facilitate further studies on PSP in neonates and young children. Blood sampling in neonates and children may represent considerable challenge due to practical issues with access difficulty, patient pain, and parental anxiety [14, 23]. Capillary sampling involving adequate skin warming is often used as a rapid and safe alternative in neonates and young children.

This study has a number of limitations. For ethical reasons, it was not feasible to obtain sequential PSP samples over a prolonged period of time in newborns and children. A subset of PSP samples were obtained in neonates enrolled in a sepsis marker study, where infection had been ruled out. Due to issues with sampling in the pediatric age group, PSP was measured in otherwise healthy newborns and in children undergoing general anaesthesia. As sampling occurred upon induction of anaesthetics for minor and elective procedures, it is unlikely that acute-phase response due to the procedure relevantly influenced PSP values in this group. The neonatal group was carefully selected to exclude patients with infection or major disease.

## Conclusions

PSP levels change significantly with age with lowest levels seen in extremely preterm babies, and highest levels observed in children. We observed a significant post-natal rise. The present study provides age-specific serum concentrations of PSP in healthy individuals which may be used as a reference data to establish optimal cut-offs in future studies on PSP.

### Key messages

- Pancreatic Stone Protein (PSP) has recently emerged as a promising marker of sepsis, and sepsis mortality in adult and neonatal intensive care studies.
- The present study provides age-specific reference values for PSP that confirm that PSP concentrations vary with patient age.
- The populational values provided in this study may serve as references for future studies to define optimal cut-off thresholds.

## Abbreviations

ICU: Intensive care unit
PCT: Procalcitonin
PSP: Pancreatic stone protein
